# HPV+ cancer cell lactate production attenuates immune response during treatment: lactate production inhibition leads to improved long-term cures

**DOI:** 10.1186/1750-9378-7-S1-P35

**Published:** 2012-04-19

**Authors:** Cathy Zhuang, Dan Vermeer Yuh-Seog Jung, Allison Haugrud, John H Lee, Keith Miskimins

**Affiliations:** 1 Sanford School of Medicine, University of South Dakota Cancer Research, Sioux Falls, SD, USA

## Background

Normal cellular metabolism is altered in cancer cells, shifting away from the TCA cycle towards glycolysis, increasing glucose consumption and lactate production. This key characteristic change in metabolism is termed the Warburg effect. Importantly, HPV 16 E7 oncoprotein alters the function pyruvate kinase type M2, increasing glucose consumption and lactate production. Clinically, increased lactate production in head and neck cancers is associated with a decreased response to therapy. Cancers with high lactate production have a poor five year survival, approximately 40% worse than similar tumors with low lactate production. Lactate has also recently been shown to disrupt functions of key immune cells (CD8 and DC’s) *in vitro*. We have recently shown that an immune response is required to clear HPV+ head and neck squamous cell carcinomas (HNSCC) *in vivo*. In this project we tested the hypothesis that lactate within the tumor microenvironment inhibits immune mediated clearance of HPV+ cancers.

## Material and methods

Experiments were completed in culture on human and mice HPV+ cancer lines, in a preclinical mouse model of HPV+ cancer and a human phase 2 clinical trial initiated.

## Results

We show that human and mouse HPV+HNSCC’s have enhanced lactate production. Inhibition of lactate with either Dichloroactetate (DCA) or Oxamate decreases tumor cell growth in colony forming assays. DCA-mediated lactate inhibition *in vivo* was well tolerated, decreased tumor lactate levels, increased tumor pH. DCA treatment by itself did not alter tumor growth significantly. However, to test whether it would enhance immune related clearance during cisplatin/radiation, studies in immune competent mice were completed and compared to identical studies in immune deficient (RAG1) mice. The studies show that inhibition of lactate production resulted in enhanced immune mediated clearance during treatment with cisplatin and radiation therapy. Furthermore, siRNA-mediated knock down of lactate dehydrogenase (LDH) confirmed the role of LDH and epithelial cell lactate production in this response. These findings show that altered metabolism and decreasing lactate in the tumor microenvironment not enhances immune clearance during therapy. Due to these finding a phase 2 clinical trial has been initiated which combines DCA with cisplatin/radiation. The initial results from the trial will be presented.

## Conclusion

Tumor produced lactate attenuates the immune clearance of HPV+ cancers. Decreasing this lactate and thus enhancing immune clearance may be very relevant for immune suppressed HIV+ individuals during therapy.

